# Complications of Hydatid Cysts of the Liver: Spiral Computed Tomography Findings

**DOI:** 10.4021/gr460e

**Published:** 2012-07-20

**Authors:** Konstantinos Alexiou, Sofoklis Mitsos, Athanasios Fotopoulos, Ioannis Karanikas, Kiriaki Tavernaraki, Fotis Konstantinidis, Peter Antonopoulos, Nikolaos Ekonomou

**Affiliations:** a Clinic Department: 1st Surgical Dept. Sismanoglion General Hospital, Athens, Greece; b Computed Tomography Unit of 1st IKA Hospital, Sismanoglion General Hospital, Greece

**Keywords:** Computed tomography, Hydatid cyst, Complications, Rupture

## Abstract

**Background:**

The purpose of this retrospective study is to evaluate the role of Spiral Computed Tomography (CT) in the detection of the complications of hepatic hydatid cysts.

**Methods:**

During a period of 8 years and after establishing the diagnosis of numerous hydatid cysts, 7 patients with complications of hydatid cysts were found. These 7 patients (5 females, 2 males, mean age 74.2 years, range 63 - 92 years) were studied. Four of them had a known medical history of hydatid disease, while all of them presented to our department as emergency cases.

**Results:**

They underwent Spiral CT which revealed the following complications of hydatid cysts: intrabiliary rupture in 2 patients, rupture into the peritoneal cavity resulting to peritonitis in 1 patient, contained rupture and secondary transdiaphragmatic thoracic rupture in 1 patient, rupture into both biliary tract and hepatic subcapsular space in 1 patient, rupture into the subcapsular hepatic space in 1 patient and secondary bacterial infection of the cyst resulting to abscess formation in 1 patient. All of these CT findings were surgically confirmed.

**Conclusions:**

CT provided a rapid and accurate diagnosis in all of these cases and proved to be a very useful preoperative imaging method.

## Introduction

Hydatid disease is a parasitic infection caused by the tapeworm Echinococcus granulosus and Echinococcus multinocularis and it is endemic usually in raising countries, but also in most developed countries in Mediteranean region, South America, Africa, etc.

The liver is the most commonly affected organ with an infestation rate of 60-75%. Specifically, the right hepatic lobe is affected in 80% of cases and the left lobe in 20%. Less common sites are the lungs (15%), spleen, peritoneum, kidneys, brain etc [1].The hydatid cyst is composed of 3 layers. The outer layer is the pericyst, an avascular layer derived from modified host tissue and inflammatory cells. The middle layer is a laminated acellular membrane and the inner layer is the germinal layer which produces the laminated membrane and the scolices. The 2 last membranes form the endocyst [1].

Hydatid disease is mostly asymptomatic and many hydatid cysts represent incidental clinical or radiological findings. Most symptomatic cysts are either complicated with rupture or secondary bacterial infection or due to their large size cause symptoms such as right upper abdominal pain, swelling and discomfort [2].

Complications of hepatic hydatid cysts are uncommon but some, in particular, can be fatal without an early and appropriate intervention and treatment [3]. The most common complication is the intrabiliary rupture of the hydatid cyst [4]. Other less common complications are the rupture of the cyst into the peritoneal cavity, rupture into the thoracic cavity through the diaphragm and toward organs of the gastrointestinal tract and the secondary bacterial infection of the cyst [5]. The purpose of this retrospective study is to evaluate the role of Spiral Computed Tomography (CT) in the detection of the complications of hepatic hydatid cysts. CT is a reliable and valuable imaging method for the study of complications of hepatic hydatid cysts with several imaging signs well established in the literature.

## Material and Methods

Within the last eight years, several cases of echinococcal hydatid cysts have been diagnosed in our department, including seven patients with complicated hydatid cyst (five females, two males, range age 63 - 92 years, mean age 74.2 years). All of them presented to our department as emergency cases, while only four of them had a known history of echinococcal disease. They underwent Spiral CT with 4 - 5 mm sections, before and after intravenous administration of contrast agent. The CT findings of these patients, which were also surgically confirmed, are presented and analyzed, as well as being correlated with clinical and laboratory findings (Table 1).

**Table 1:** Characteristics of Patients

Patients							
No	1	2	3	4	5	6	7
Sex and Age	F 92	M 70	F 80	F 63	F 86	M 65	F 64
Known history of hydatid disease	Yes	No	Yes	Yes	No	Yes	No
Clinical features	Acute right upper abdominal quadrant pain	Acute right upper abdominal quadrant pain; Fever	Diffuse abdominal pain Fever Abdominal wall contraction Rebound sensitivity	Diffuse abdominal pain Right thoracic pain Fever; Dyspnea	Acute right upper abdominal quadrant pain with lumbar radiation	Acute right upper abdominal quadrant pain; Fever	Acute right upper abdominal quadrant pain
Laboratory findings	Eosinophilia Increased amylase serum Increased direct bilirubin serum	Eosinophilia Increased direct bilirubin serum	Increased WBC count	Eosinophilia	Mild leukocytosis	leukocytosis	Increased direct bilirubin serum
Treatment	Surger	Surgery	Surgery	Surgery	Surgery	Surgery	Surgery
Outcome	Improvement	Improvement	Death	Improvement	Improvement	Improvement	Improvement

## Results

**Figure 1:**
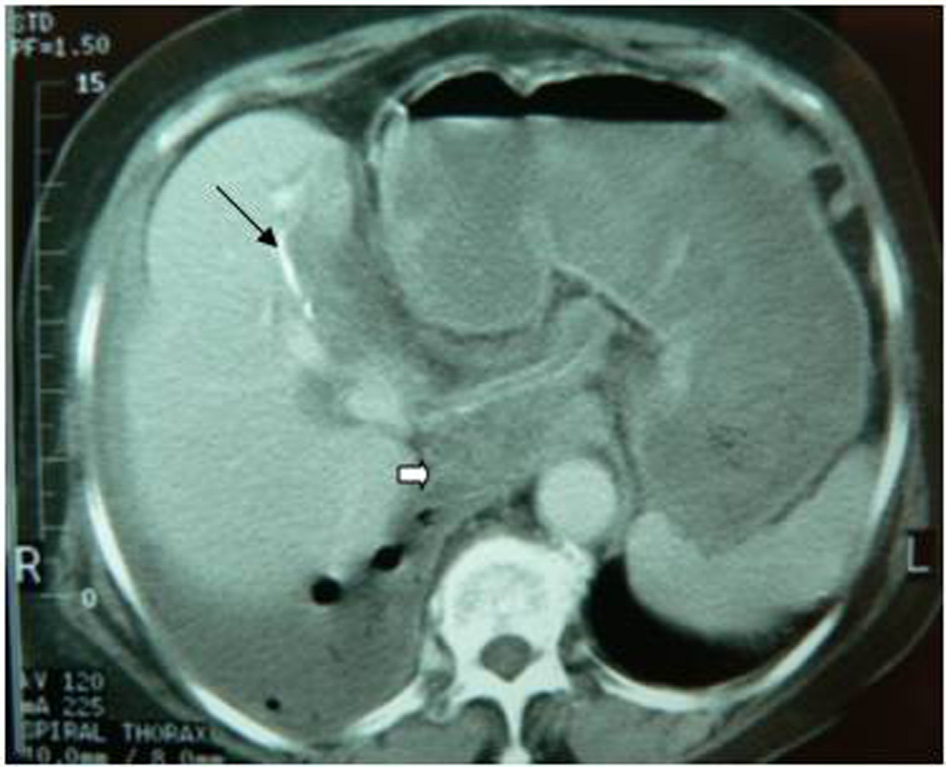
Transdiaphragmatic thoracic rupture. It is observed the calcified hydatid cyst wall (black arrow), daughter cysts (white arrow) and pleural effusion with presence of air.

**Figure 2:**
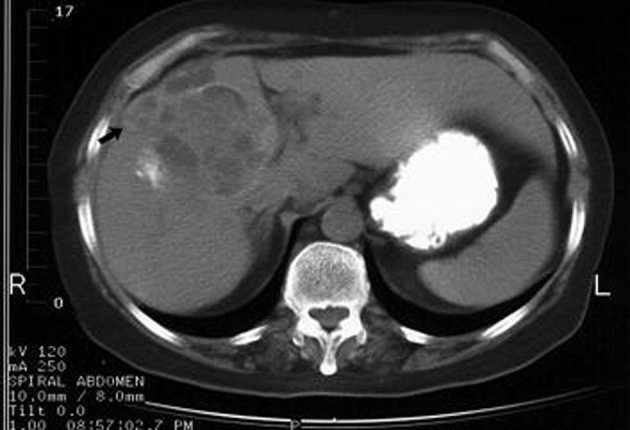
Rupture into biliary tract and subcapsular hepatic space. Change in architecture of the cyst, presence of fluid collection and daughter cysts in the subcapsular hepatic space (arrow) and dilatation of biliary radicles.

All the patients underwent Spiral CT, which revealed the following complications of hydatid cysts: Two patients had intrabiliary rupture, rupture into the peritoneal cavity resulting to peritonitis had been diagnosed in 1 patient, contained rupture and secondary thoracic rupture in 1 patient (Fig. 1), rupture into both biliary tract and hepatic subcapsular space in 1 patient (Fig. 2), rupture into the subcapsular hepatic space had been noticed in 1 patient and finally, secondary bacterial infection of the cyst resulting to abscess formation in 1 patient. All the CT findings were surgically confirmed.

## Discussion

The complications of hepatic hydatid cysts are generally rare and they include two main categories: rupture and secondary bacterial infection [1]. Rupture of the hydatid cyst is more frequent, occurring in 20-50% of cases, while the secondary bacterial infection appears in only 5-8 % of cases [6]. Several theories have been proposed in concern of the mechanism of the rupture, including the degeneration of the parasitic membranes due to chemical or host defense mechanisms, the ageing of the hydatid cyst and trauma [5].

Modern imaging methods, such as ultrasound, computed tomography and magnetic tomography, have now given the option of safe diagnosis of hydatid disease and its complications. Despite the fact that it is supported that ultrasound and ERCP are enough for the preoperative diagnosis of intrabiliary rupture of hepatic echinococcal cyst [7], the ERCP is not available in all hospitals and mainly used therapeutically. Computed tomography is a method of choice for diagnosis of hydatid disease and possible complications, because it is a quick, painless, noninvasive and reliable method [6, 8].

Three types of rupture have been described: contained, communicating and direct. Contained rupture occurs when the endocyst ruptures and hydatid fluid escapes into the space between the pericyst and the endocyst resulting to the collapse of the endocyst [5]. This collapse of the endocyst can be recognized on CT which shows detached undulating memebranes inside the hydatid cyst without the reduction in the cyst size. This diagnostic sign is called the “waterlilly sign” or “snake or serpent sign” because of its appearance [5, 9, 10].

Communicating rupture is the most common type of rupture, appearing in 44-64% of cases and its occurs when the content of hydatid cyst evacuates into the biliary radicles that have been incorporated by the pericyst [11]. The communating type of rupture results from contained rupture and it is subdivided into two groups depending on the size of communication [11]. In the first subtype, which is more frequent, the communication concerns in small biliary radicles and it is established by small fissures or bile-cyst fistulas [10]. The second subtype is characterized by a wide perforation of the hydatid cyst into a main biliary radicle [11]. In both cases hydatid fluid, hydatid sand and occasionally daughter cysts can be discharged into the biliary tree.

Intrabiliary rupture of a hepatic hydatid cyst, although a rare entity, is the most common complication of hepatic hydatid cyst with a reported incidence of 3-17% [4, 12]. Rupture occurs mainly into the right hepatic duct (55-60% of cases) and less frequently into the left duct (25-30%) and the confluence or gall bladder [13, 14]. The clinical manifestations of intrabilliary rupture include right upper abdominal pain, obstructive jaundice, fever, cholangitis or sepsis and even death if the complication is not in early stages recognized [15]. Therefore, a rapid and accurate pre-operative diagnosis is essential. CT is an imaging method which provides such diagnosis based on specific findings [8, 16]. The discontinuity of the calcified or not hydatid cyst wall, the change in architecture of the cyst and the dilatation of the biliary tree with air or air-fluid level inside the cyst and the dilatation of the biliary tree with air or containing high density linear structures, representing hydatid material are characteristic imaging findings which can lead to the final diagnosis of the rupture [5, 11]. Generally, due to the fact that most communating ruptures occur through small fissures, it is very difficult to determine on CT the exact site and location of rupture. Therefore, CT examination must be performed with slice thickness of maximum 3 - 5 mm. In our study, focal herniation of the cyst wall was an indicative finding of the rupture site.

The last type of rupture of a hepatic hydatid cyst, is the direct rupture which occurs when both the endocyst and pericyst are torn so that the content of the cyst escapes into the peritoneal cavity, the thoracic cavity through the diaphragm, the mediastinum or occasionally a hollow organ such as the colon [5, 11].

Direct rupture of the hepatic hydatid cyst into the peritoneal cavity can be detected on CT by findings such as detached membranes, reduction of the cyst size, cyst wall discontinuity and change in the architecture of the hydatid cyst which are the general imaging findings of rupture [5, 17]. The presence of daughter cysts inside the peritoneal cavity can also be diagnosed by CT [17, 18]. Although very rare, this complication can be fatal without the appropriate management, as the hydatid cyst material causes allergic shock, several chemical reactions in the peritoneal cavity and can result to chemical and eventually secondary bacterial peritonitis. In that case, apart from the imaging findings of rupture, fluid effusions in the peritoneal cavity, fat standing and opacification, dilatation of mesenteric vessels and thickening of the peritoneum may also be detected on CT as a result of peritonitis. Another complication of direct rupture of hepatic hydatid cyst into the peritoneal cavity is the implantation of scolices in several organs leading to a condition called “metastatic hydatidosis” [5].

Direct rupture of hepatic hydatid cyst can also be limited in the hepatic subcapsular space in a way that the hydatid material will not escape into the peritoneal cavity due to the protective effect of Glisson’s capsule. This does not allow the free spillage of the cyst’s content into the peritoneal cavity and therefore limits the rupture, as observed in 2 of our cases.

The transdiaphragmatic intrathoracic rupture of a hepatic hydatid cyst is another complication which occurs in 0.6-16% of patients with hepatic hydatid disease [17, 19]. The bare area of the liver, specifically the posterior segments of the right hepatic lobe, is the most common route of transdiaphragmatic migration of hydatid material due to lack of peritoneal covering in this area [20]. The presence of air inside the hydatid cyst, when observed on CT, is strongly indicative of communication with the bronchial tree. Pleural effusions, lung consolidation and atelectasis due to chemical reactions from the migration of hydatid material into the lungs are common imaging findings on CT indicating the thoracic involvement [21, 22].

Secondary bacterial infection of the hepatic hydatid cyst is a very rare complication due to the avascularity of the pericyst and the lack of connection between the endocyst and the host vascular system [5].

Essential prerequisite for the bacterial infection of the cyst is the rupture of both pericyst and endocyst, so bacteria can enter the cyst [5]. The presence of air forming an air fluid level inside the cyst, while the size of the cyst remains intact, is a finding often observed on CT in such cases and may indicate the infection of the hydatid cyst, although it may be observed in other cases as well [9]. The clinical status of the patient and specific laboratory data will contribute to the final diagnosis in cases of infection of the cyst which acts as a hepatic abscess.

In conclusion, although a rarity, complications of hepatic hydatid disease can prove to be life-threatening and therefore should be early and accurately recognized. Specific imaging findings on Spiral CT allow definite diagnosis and prompts operative intervention.
